# Do Functional Movement Screens Predict Body Composition Changes after Resistance Training?

**DOI:** 10.1055/a-2556-4182

**Published:** 2025-06-24

**Authors:** Jared Rosenberg, Jytosna Natarajan, David J Carenter, Chris Peluso, Christie Hilton, Colin E. Champ

**Affiliations:** 1 Kinesiology Department, SUNY Cortland, NY, United States; 2 Drexel University College of Medicine, Philadelphia, PA, United States; 3 Department of Radiation Oncology, Wellstar Paulding Medical Center, Hiram, GA, United States; 4 Allegheny Health Network Exercise Oncology and Resiliency Center, Pittsburgh, PA, United States; 5 Department of Medical Oncology, Allegheny Health Network, Pittsburgh, PA, United States; 6 Department of Radiation Oncology, Allegheny Health Network, Pittsburgh, PA, United States

**Keywords:** functional movement screen, resistance training, breast cancer

## Abstract

Although the function movement screen (FMS) has been widely used in the general population, no study to date has used the FMS as a preparticipation screen for individuals with breast cancer (BC) engaging in an exercise regimen. Even though individuals with BC are anthropometrically similar to individuals without cancer, the lack of studies assessing the FMS in individuals with BC may potentially hinder exercise prescription. Therefore, we aim to examine the relationships of the FMS score to anthropometric biomarkers in individuals with BC before undergoing an exercise regimen. One-hundred and twelve women with BC underwent a thrice-weekly three-month dose-escalated exercise regimen utilizing multi-joint compound movements and linear progression balanced with resistance training volume to elicit hypertrophy. FMS score and anthropometric markers were assessed pre- and post-intervention. With significance set at p≤0.05, baseline FMS scores correlated significantly with all anthropometric markers, and was similar to previous studies published in non-cancer populations. However, baseline FMS scores were not associated with changes in anthropometric markers, from pre- to post-intervention. While the baseline FMS score was not associated with changes in anthropometric markers, the similar correlation found in our study compared to previous studies suggest that the FMS can be used as a preparticipation in individuals with BC to help guide the exercise regimen. Future studies designed to elicit weight loss in individuals with BC should assess whether the baseline FMS score is predictive of anthropometric changes.

## Introduction

As exercise, activity levels, muscle mass, and decreased fat mass are associated with improved survival in women undergoing treatment for breast cancer, efforts are underway to include resistance training as a routine part of breast cancer (BC) treatment 1 . However, efforts to maximize changes in body composition while optimizing safety are important in this patient population. There is currently no reliable and quantifiable test that measures mobility and can be utilized for research and to quantify mobility changes during progression of exercise prescriptions. The functional movement screen (FMS) is an exercise pre-participation screen that can help guide the exercise prescription to maximize the benefits of exercise, such as muscle hypertrophy, fat loss, and improved strength, while minimizing potential injury risk 2 3 4 . It is comprised of seven movements, with each movement scored from 0 to 3; a score of 3 indicates perfect movement without compensation, while 0 represents pain during the movement. Thus, the total score can range from 0 to 21 5 6 . The FMS can uncover compensatory movement during basic movement patterns. Compensatory movement patterns may predispose individuals to injuries. As such, the FMS has been shown to predict which individuals are at a heightened risk of injury during certain movement patterns 6 .

Previous literature suggests that, in middle and older aged healthy adults, the FMS score correlates negatively with body mass index (BMI), percent body fat, and age, and positively correlates with activity levels 7 8 . Individuals with BC are anthropometrically similar to individuals without BC, except for potential mobility issues from surgical treatment and radiation therapy 1 . Thus, it may be reasonable to assume that the relationship of the FMS to BMI, percent body fat, age, and activity levels would be similar in individuals with BC compared to the general population. However, the relationship of FMS score to BMI, percent body fat, age, and activity levels has not been tested in individuals with BC. Furthermore, it remains unknown whether individuals undergoing an exercise regimen with higher FMS scores lose more weight than those individuals with lower FMS scores. This represents a critical gap in our understanding of implementing a proper exercise regimen to maximize the efficacy and minimize risk of exercise programs, particularly as we dose-escalate exercise programs in individuals treated for BC 9 .

Therefore, the aims of this study in women with BC were to 1) assess the relationship of FMS to BMI, percent body fat, age, and activity levels; and 2) assess if individuals with higher FMS scores lost more weight and/or percent body fat, than individuals with lower FMS scores from pre- to post-intervention.

We hypothesized that 1) the FMS score would have a strong relationship with BMI, percent body fat, age, and activity levels; and 2) individuals with higher FMS score would lose more weight and a greater percent of body fat compared to individuals with lower FMS scores.

## Methods

### Participants

We reviewed all participants of EXERT-BC, EXERT-BCN, and EXERT-C, which were three institutional review board-approved prospective exercise studies in women aged 20–89 with biopsy-proven ductal carcinoma in situ (DCIS) or BC. All participants signed consent forms prior to any testing. Participants in each study were required to be able to get up and down from the ground, squat their body weight, and participate in a group exercise regimen. Individuals with severe arthritic, joint, cardiovascular, and/or musculoskeletal condition deemed unsafe to engage in resistance training were excluded. Participants actively receiving systemic cytotoxic chemotherapy were excluded from the studies, while participants treated with radiation, anti-estrogen and targeted systemic therapies were allowed. Participants were screened by study personnel at the time of oncologic consultation or a follow-up visit.

Recruitment occurred between September 15, 2022, and October 17, 2023, at the Allegheny Health Network (AHN) departments of surgical, medical, and radiation oncology, along with the AHN Cancer Institute Exercise Oncology and Resiliency Center (EOC). Consent was obtained for each participant prior to enrollment in the study. The studies are registered at ClinicalTrials.gov (NCT05747209, NCT05978960, and NCT06083324).

### Functional Movement Screen

Prior to initiation of the exercise regimen and at completion, each participant underwent an FMS assessment performed by expert personnel. The physician who screens all patients is level I and II FMS certified and has performed the test in over 1 000 individuals. The other exercise physiologist who performs the test was trained by him and showed proficiency in the FMS under close observation before being able to implement it. Both practitioners observe each other weekly during the test to enhance accuracy and proficiency. Each participant performed 7 movements (deep squat, hurdle step, in-line lunge, shoulder mobility, active straight-leg raise, trunk stability push-up, and rotary stability), which were scored from 0 to 3. A score of 0 was given if there was any pain associated with the movement by the participant. A score of 1 was given if the individual could not perform the movement. A score of 2 was given if the movement was performed with compensatory movements, and a score of 3 was given if the movement was completed without compensatory movements 10 . The composite score of all seven movements was summed, with the absolute lowest score of 0 and highest of 21. For the unilateral movements (hurdle step, inline lunge, shoulder mobility, active straight-leg raise, and rotary stability), each movement was scored independently on the left and right sides of the body. If a movement was scored differently from one side to the other, the lower of the two scores was used in the composite score 5 11 .

### Exercise intervention

The exercise regimen included a combination of compound movements focusing on closed kinetic chain movements (CKC), utilizing linear progression and following guidelines from the National Strength and Conditioning Association (NSCA). Activation and reset exercises focused on mobility, muscle activation, and range of motion were performed prior to each workout to reduce the risk of injury. Each workout provided full body resistance training focusing on the basic movement patterns of push, pull, hip hinge, squat, and core activation. To maximize safety, each individual exercise workout progressed from high intensity, CKC, compound, athletic movements, such as squats and deadlifts, to low intensity, more isolated focused exercises throughout the workout 12 . The entire program lasted 12 weeks, with each exercise session ranging from 45–60 minutes. Lastly, there was a two-week ramp-up period at the start of the program, and weights lifted utilized a combination of repetition speed, number “left in the tank,” and rating of perceived exertion.

The study took place at the Allegheny Health Network Cancer Institute’s Exercise Oncology and Resiliency Center. The center is a state-of-the-art, 3 000-square-foot exercise and research facility where the exercise regimens are created and monitored by Certified Strength and Conditioning Specialists (CSCS) and a medical doctor, utilizing exercise principles to increase strength, conditioning, performance, and overall health.

Exercise class attendance was recorded and planned missed days were able to be performed remotely if the individual had access to similar workout equipment. This was allowed only after the first month of the regimen. Exercises were progressed or regressed around specific core movement patterns (push, pull, hip hinge, squat, and core) based on participant ability. For example, if an individual was unable to perform a bodyweight split squat, they would be assisted in the movement until they could progress to the weighted lift. Weight lifted, repetitions, sets, and notes were recorded.

### Experimental design

All participants were enrolled in a three-month, thrice weekly dose-escalated exercise regimen utilizing multi-joint compound movements and linear progression balanced with resistance training volume to elicit hypertrophy, as previously described 1 13 . The following study includes data from three different cohorts: EXERT-BC (n=40), EXERT-C (n=29), and EXERT-BCN (n=43). All groups underwent the same intervention, except for individuals in the EXERT-BCN cohort, who underwent an additional nutrition intervention requiring nutrient dense food sources high in vitamins, minerals, and nutrients. They were encouraged to limit processed foods, sugar, bread, pasta, and other simple carbohydrates, and to eat plenty of colorful and non-starchy vegetables. They were also advised to avoid snacking between meals, cook most/all meals, eat with family and friends, avoid eating food in the car or on the run, and focus on whole foods that require preparation. The goal of protein consumption was at least 1.3–1.8 g/kg per day.

### Body composition and resting metabolic rate

Prior to initiation and at completion of the exercise regimen, each participant underwent body composition analysis via an InBody 970 bioimpedance analysis (BIA) machine (InBody Co., Seoul, South Korea). Body composition analysis included total body fat (lbs.) and total muscle mass (lbs.). Additionally, an ultrasound (US) was performed to measure percent body fat, fat-free mass (FFM), and resting metabolic rate (RMR) 14 , which was calculated utilizing Body Metrix software (BodyMetrix, Brentwood, CA, USA).

### Strength and load lifted

Prior to initiation and at completion of the exercise regimen, each participant underwent bilateral grip strength assessments with each arm at the neutral position, utilizing a Jamar Hand Dynamometer grip strength measurement device. Load was calculated continuously throughout the regimen by multiplying weight lifted (lbs.) by repetitions and sets. These calculations were done at the fourth, eighth, and the final week of the exercise regimen to ensure dose escalation. Split squat, trap bar deadlift, incline dumbbell bench press, and birddog row were compared as these encompass squat, hip hinge, push, and pull movement patterns.

### Activity levels

Preceding the initiation of the exercise regimen, each participant completed a Godin Leisure-Time Exercise Questionnaire 15 . The questionnaires were completed at the end of their exercise regimen as well.

### Statistical analysis

Sample size was determined a priori for each cohort using G*Power. Using pilot data from our lab, we observed a moderate effect size (Cohen’s d=0.57) for the change in percent body from pre-to post-intervention. Using this effect size, an alpha set at 0.05, and power set at 0.95, the power analysis determined a total of 37 participants would be needed. Therefore, we aimed to recruit approximately 37 participants for each cohort (EXERT-BC, EXERT-C, and EXERT-BCN). Given our previous pilot data, we did not anticipate any drops, and thus we did not consider dropouts in the power analysis.

Spearman’s correlation was used to examine bivariate relationships between FMS scores and BMI, percentage body fat, age, and activity level at baseline. Dependent t-tests were used to assess the change from pre- to post-intervention for all continuous variables. Linear regression was performed to assess whether baseline FMS score was associated with weight loss during the three-month intervention. Data that did not meet the assumptions for normality were log _10_ -transformed; untransformed data are presented for ease of interpretation. For variables that were negative (i. e., weight loss), a constant was added to make the variable a positive integer, thus the variable could then be log _10_ -transformed. The regression models were run with the cohorts individually, and subsequently with all the cohorts combined. Data were analyzed using SPSS 28.0 (IBM Corp., Armonk, NY, USA) for the analysis of descriptive statistics, comparison of means, correlations, and regressions, with significance set at p≤0.05. Data are mean±SEM.

## Results

### Patient characteristics

Baseline participant characteristics are shown in Table 1 . At baseline, 112 women with BC were assessed and completed one of three protocols. Eighteen women were undergoing systemic targeted treatment, 29 were receiving radiation therapy, and 91 were on anti-estrogen therapy. No difference was found between the three cohorts for the percentage of individuals undergoing chemotherapy, radiation, or anti-estrogen therapy ( **Supplemental Table 1** ). Of the 112 participants, only one injury was noted, a knee injury that resulted in missing a planned workout. An average of 3.3 missed workouts per participant were recorded, with 26 individuals not missing a single workout during the duration of the 3-month trial (data not shown).

**Table:** **Table 1** Descriptive characteristic from pre- to post-intervention in all individuals who completed the trial.

Descriptive (n)	Pre-intervention	Post-intervention	Cohen’s d	*P* -value
BMI (112)	29.3±0.6	28.8±0.6	0.49	<0.001
Body fat (%) (112)	35.0±0.6	32.1±0.6	0.43	<0.001
Muscle mass (lbs.) (112)	56.4±0.8	57.4±0.8	0.11	<0.001
FFM (%) (112)	29.5±0.4	30.5±0.4	0.21	<0.001
RMR (111)	1,441.4±15.2	1,474.1±15.9	0.20	<0.001
FMS (110)	10.2±0.3	12.5±0.3	0.75	<0.001

BMI, body mass index; CI, confidence interval; FFM, fat free mass; RMR, resting metabolic rate; FMS, functional movement screen. Mean difference is difference from post- to pre-intervention; P-value is pre- vs. post-intervention; data is mean±SEM.

### Baseline assessment

**Fig. 1:**
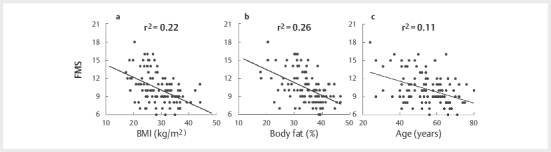
Correlation of FMS to BMI ( **a** ), body fat % ( **b** ), and age ( **c** ).

**Fig. 2:**
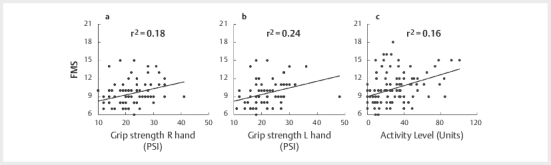
Correlation of FMS to right hand grip strength ( **a** ), left hand grip strength ( **b** ), and activity level ( **c** ).

At baseline, no difference was noted in age, BMI, percent body fat, FFM, RMR and FMS score between all three different cohorts ( **Supplemental Table 2** ). The FMS score negatively correlated with BMI (r=–0.466 [–0.603, –0.301], p<0.001), percent body fat (r=–0.512 [–0.641, –0.356], p<0.001), and age (r=–0.334 [–0.494, –0.153], p<0.001) ( Fig. 1 ). Furthermore, baseline FMS correlated positively with activity level(r , 0.395 [0.221, 0.545], p<0.0001), right hand grip strength (r=0.428 [0.258, 0.573], p<0.001), and left hand grip strength (r=0.493 [0.333, 0.626], p<0.001) ( Fig. 2 ).

Moreover, activity level also negatively correlated with BMI and percent body fat (r=–0.207 and –0.203, respectively; both p≤0.01). Therefore, we decided to assess the relationship of FMS to BMI and percent body fat while controlling for activity level. Both the relationship of the FMS to BMI and percent body fat remained significantly negatively correlated (r=–0.440 and –0.481, respectively; both p<0.001).

### Longitudinal assessment

From pre- to post-assessment, significant decreases in BMI and percent body fat were observed. Furthermore, significant increases in FMS score, FFM, and RMR were noted pre- to post-assessment (all p<0.05) ( Table 1 ).

Linear regression revealed that baseline FMS score was not significantly associated with changes in BMI ( Table 2 ) or percent body fat ( Table 3 ). When BMI and body fat were calculated as percentages of respective baseline values, the baseline FMS score was still not significantly associated with changes in BMI ( Tables 2 and 3 ). Similarly, changes across pre- and post-intervention FMS scores were not associated with changes in BMI or percent body fat ( Tables 2 and 3 ).

**Table:** **Table 2** Regression models for change in BMI.

	Model	Unstandardized Coefficients		Standardized Coefficients	R Square Change	t	Sig.
		β	Std. Error	Beta			
**1**	Constant	0.504	0.103			5.341	<0.001
	FMS score	0.094	0.103	0.087	0.008	0.916	0.362
**2**	Constant	1.138	0.186			6.129	<0.001
	FMS score	–0.313	0.186	–0.159	0.025	–1.689	0.094
**3**	Constant	0.646	0.039			16.369	<0.001
	Change in FMS score	–0.007	0.056	–0.013	0.0001	–0.130	0.897
**4**	Constant	0.517	0.109			4.724	<0.001
	FMS score	0.004	0.003	0.108	0.039	1.106	0.082

Model 1 is the association of baseline FMS score to change in BMI. Model 2 is the association of baseline FMS score to change in the percent BMI, relative to baseline. Model 3 is the association of the change in FMS score, relative to baseline, to change in BMI. Model 4 is the association of baseline FMS score to change in BMI while adjusting for missed workouts.

**Table:** **Table 3** Regression models for change in BF%.

	Model	Unstandardized Coefficients		Standardized Coefficients	R Square Change	t	Sig.
		β	Std. Error	Beta			
**1**	Constant	1.154	0.203			5.698	<0.001
	FMS score	–0.146	0.202	–0.069	0.005	–0.724	0.471
**2**	Constant	1.521	0.151			10.093	<0.001
	FMS score	0.035	0.151	0.022	0.001	0.234	0.815
**3**	Constant	1.019	0.077			13.325	<0.001
	Change in FMS score	–0.009	0.108	0.008	0.0001	0.088	0.930
**4**	Constant	1.004	0.213			4.898	<0.001
	FMS score	–0.013	0.007	0.187	0.032	1.923	0.064

Model 1 is the association of baseline FMS score to change in BF. Model 2 is the association of baseline FMS score to change in the percent BF relative to baseline. Model 3 is the association of the change in FMS score relative to baseline to change in BF. Model 4 is the association of baseline FMS score to change in BF while adjusting for missed workouts.

Furthermore, a hierarchical multiple regression revealed that total days missed did not improve the association of baseline FMS for change in BMI ( Table 2 ). However, a hierarchical multiple regression revealed that total days missed did improve the association of baseline FMS for change in body composition, although significance was not fully reached ( Table 3 ).

Lastly, when assessing each cohort individually, baseline FMS score was not associated with change in BMI or percent body fat, even when expressed as a percentage compared to baseline ( Tables 2 and 3 ).

## Discussion

Our current study revealed that 1) baseline FMS score negatively correlated with BMI, percent body fat, and age; 2) the baseline FMS score positively correlated with activity level; and 3) the baseline FMS score was not associated with changes in BMI or body composition. These findings suggest that, while the FMS can be used to guide exercise prescription for individuals with BC, the baseline FMS is not associated with changes in body weight or body composition after undergoing an exercise regimen utilizing intense resistance training.

Improved body composition is of critical significance for women with BC. Individuals with obesity are at risk of worse outcomes after treatment for BC 16 17 , emphasizing fat reduction to potentially improve BC outcomes. However, during weight loss, as much as 38% of weight can be lost from muscle tissue, jeopardizing body composition 18 . For individuals with BC, this may be particularly harmful, as low muscle mass is associated with higher mortality rates 19 . Further highlighting the importance of muscle mass for individuals with BC, it is estimated that cachexia accounts for up to 20% of cancer-related deaths 20 . Resistance training is the most potent non-pharmacological intervention to increase muscle mass 21 . Our clinically relevant findings of an increase in muscle mass, despite a loss in body mass, should be used as a proof of concept that an exercise regimen focused on closed chain, intense resistance training can be implemented safely and effectively in individuals with BC, even in those with poor initial mobility. Such results are encouraging and translatable to the general BC population as entrance criteria for these studies were broad. Additionally, no injuries were reported in either study, besides one episode of transient knee pain, further supporting progression during resistance training for these individuals.

The present study was the first of its kind to assess the FMS as a pre-participation exercise screen for individuals with BC. Previously, we have shown that a majority of exercise oncology studies observing women with BC use mostly open chain kinetic exercises 22 . Safe and effective implementation of CKC movements into an exercise intervention may have been limited by the lack of a pre-participation movement screen for individuals with BC. Our findings suggest that the FMS can help exercise practitioners guide these closed chain, intense full-body exercise sessions to maximize the potential benefit and safety. Although our decision to FMS-screen all participants was done a priori, we used it to guide and modify each individual’s exercise regimen, if needed. This may ultimately be responsible for the low injury rates. Moreover, the implementation of FMS score-guided exercise modification may have led to an enjoyable and challenging exercise experience, as before the intervention only 12 individuals had any prior experience with resistance training, and afterwards, 54 planned on continuing resistance training (data not shown).

Our data further support the utility of the FMS as a useful tool to guide exercise prescription. We found that the correlation of FMS score with BMI, percent body fat, and activity level was comparable to those reported among non-cancer individuals 7 23 . Additionally, these correlations remained significant after accounting for activity level. Similar findings and implications were found by Perry et al. in middle-aged adults without cancer 24 . Thus, our data suggest that the FMS screen can be used for individuals with BC. Such implementation could potentially result in an exercise intervention that includes more CKC movements, which has the potential to result in profound anthropometric and metabolic changes.

Another key finding from this study is that the baseline FMS score was not associated with weight loss or reductions in percent body fat. Similarly, the change in FMS score, from pre- to post-intervention, was also not associated with weight loss or reductions in percent body fat. However, when running a hierarchical regression model with accounting for workouts missed, a trend towards significance was found for the association of baseline FMS score to change in body composition. Thus, potentially suggesting that for individuals who want to improve body composition, baseline movement capabilities are not as impactful as total volume of exercise. It should be noted that, while a significant amount of weight loss did occur, this study was not designed to elicit weight loss, and future studies should examine if a higher baseline FMS score is a predictor of weight loss.

The strengths of the present study include 1) a first-time assessment of the relationship of FMS to BMI, percent body fat, age, and activity levels among BC patients; 2) a first-time evaluation assessing the predictive value of FMS score to changes in body weight and composition; and 3) a large group with a wide range of ages and varying stages of breast cancer with varying treatments. While the lack of uniformity in cancer stage and treatment may be perceived as a limitation, our participants are more representative of the BC population, providing more generalizable findings. One potential limitation is that these findings may not be translatable without the guidance of CSCS and FMS trained individuals. Without proper training, implementing the FMS to guide exercise prescription may not be feasible for an individual who is exercising independently. While modern bioimpedance analysis utilizing multicompartment measurements is considerably more accurate than prior ones, it can still be affected by hydration status, providing further limitations on the data. We also utilized BMI for some of our calculations, which does not account for muscle mass versus fat mass.

## Conclusion

In summary, our study reveals that in women with BC, the FMS 1) correlates with anthropometric markers, grip strength and activity levels; and 2) does not predict changes in anthropometric markers during a three-month exercise program.

Our data suggest that the FMS can be used in individuals with BC to optimize their exercise regimen. However, baseline FMS values are not associated with changes in pre- and post-intervention changes in body fat or BMI and should not be used to predict changes in anthropometric markers during an exercise intervention. These data support utilizing FMS to guide safe dose escalation of intense CKC movements for BC patients. Future studies should assess if baseline FMS scores can predict changes in strength-based measures, such as grip strength.
