# Supplementary material for: Do Functional Movement Screens Predict Body Composition Changes after Resistance Training?
Source: Sports Med Int Open. 2025 Jun 24;9:a25564182. doi: 10.1055/a-2556-4182 (PMC12265395; doi:10.1055/a-2556-4182)
Supplement: Supplementary file 1 — Supplementary Material [file 10-1055-a-2556-4182-09-2024-0257-cs.pdf]

Table Sup 1. Descriptive characteristic from pre- to post-intervention in all individuals who completed the BC trial.

Descriptive	N	Pre-intervention	N	Post-intervention	*P*-value
BMI	40	29.0 ± 1.0	40	28.8 ± 1.0	0.075
Body fat (%)	40	36.2 ± 1.0	40	32.4 ± 1.0	<0.001
Muscle mass (lbs.)	40	56.4 ± 1.1	40	57.5 ± 1.1	<0.001
FFM (%)	40	28.9 ± 0.7	40	30.6 ± 0.7	<0.001
RMR	40	1,422.8 ± 22.8	40	1,476.8 ± 26.2	<0.001
FMS	40	9.5 ± 0.4	40	12.1 ± 0.5	<0.001

BMI, body mass index; FFM, fat free mass; RMR, resting metabolic rate; FMS, functional movement screen. P-value is pre- vs. post-intervention; data is mean ± SEM.

Descriptive characteristic from pre- to post-intervention in all individuals who completed the C trial.

Descriptive	N	Pre intervention	N	Post intervention	*P*-value
BMI	28	28.8 ± 1.2	28	28.3 ± 1.1	0.018
Body fat (%)	29	33.0 ± 1.4	29	31.4 ± 1.3	0.033
Muscle mass (lbs.)	29	55.2 ± 1.6	29	56.0 ± 1.5	0.070
FFM (%)	29	28.8 ± 0.8	29	29.0 ± 0.7	0.393
RMR	28	1,407.7 ± 27.1	28	1,415.8 ± 25.9	0.369
FMS	29	11.0 ± 0.5	29	12.8 ± 0.7	<0.001

BMI, body mass index; FFM, fat free mass; RMR, resting metabolic rate; FMS, functional movement screen. P-value is pre- vs. post-intervention; data is mean ± SEM.

Descriptive characteristic from pre- to post-intervention in all individuals who completed the BCN trial.

Descriptive	N	Pre intervention	N	Post intervention	*P*-value
BMI	43	29.9 ± 0.9	43	29.3 ± 0.9	<0.001
Body fat (%)	43	35.2 ± 0.8	43	32.2 ± 0.8	<0.001
Muscle mass (lbs.)	43	57.4 ± 1.4	43	58.2 ± 1.3	0.004
FFM (%)	43	30.6 ± 0.8	43	31.4 ± 0.8	0.005
RMR	43	1,481.2 ± 27.2	43	1,509.8 ± 27.5	0.002
FMS	41	10.4 ± 0.4	41	12.7 ± 0.5	<0.001

BMI, body mass index; FFM, fat free mass; RMR, resting metabolic rate; FMS, functional movement screen. P-value is pre- vs. post-intervention; data is mean ± SEM.

.

*Body mass index (BMI) and muscle mass via bioimpedance analysis. Body fat, fat-free mass, and resting metabolic rate via ultrasound.

Descriptive characteristic from participants in the EXERT-BC, EXERT-BCN, and EXERT-C studies.

Descriptive	Trial (N)	Mean	Trial (N)	Mean	Trial (N)	Mean	P-value
Age	BC (40)	56.2 ± 1.6	C (29)	54.9 ± 2.7	BCN (43)	53.9 ± 1.6	0.651
BMI	BC (40)	29.0 ± 1.0	C (29)	28.8 ± 1.2	BCN (43)	29.1 ± 1.0	0.720
Body fat (%)	BC (40)	36.2 ± 1.0	C (29)	33.0 ± 1.4	BCN (43)	35.2 ± 0.8	0.077
FFM (%)	BC (40)	28.8 ± 0.7	C (29)	28.8 ± 0.8	BCN (43)	30.6 ± 0.8	0.391
RMR	BC (40)	1422.2 ± 22.8	C (29)	1415.8 ± 27.4	BCN (43)	1481.2 ± 27.2	0.168
FMS	BC (40)	9.5 ± 0.4	C (29)	11.0 ± 0.5	BCN (43)	10.3± 0.4	0.069
Activity level	BC (40)	24.8 ± 3.7	C (29)	25.2 ± 4.1	BCN (43)	30.1 ± 3.2	0.489

BMI, body mass index; FFM, fat free mass; RMR, resting metabolic rate; FMS, functional movement screen. P-value is difference amongst the studies; data is mean ± SEM.

Table Sup 2. Descriptive baseline characteristic from participants in the EXERT-BC, EXERT-BCN, and EXERT-C studies combined.

Physical Characteristics	N	Mean
Age (years)	112	55.0 ± 1.1
BMI (kg/m^2)	112	29.3 ± 0.6
Body fat (%)	112	35.0 ± 0.6
FFM (%)	112	29.5 ± 0.4
RMR	112	1443.2 ± 15.1
FMS	112	10.2 ± 0.2

BMI, body mass index; FFM, fat free mass; RMR, resting metabolic rate; FMS, functional movement screen. Data is mean ± SEM.

Frequencies of different treatment received by each study.

Study	AET during (Y/N)	Radiation (Y/N)	Chemo (Y/N)	P-value
BC	34/6	12/28	6/34	NS
BCN	37/6	32/11	6/37	NS
C	20/8	6/22	6/22	NS

AET, anti-estrogen therapy. P-value comparison of the prevalence across each study.
